# Telomerase and estrogen-sensing activities are essential for continued mammary growth *in vivo* but dispensable for “reprogramming” neural stem cells

**DOI:** 10.18632/aging.100985

**Published:** 2016-06-24

**Authors:** Andrea L. George, Corinne A. Boulanger, Gilbert H. Smith

**Affiliations:** ^1^ Basic Research Laboratory, Center for Cancer Research, National Cancer Institute, National Institutes of Health, Bethesda, MD 20892, USA

**Keywords:** telomeres, mammary, senescence, stem cells

## Abstract

It has been proposed that the erosion of telomere length is a limiting factor in replicative capacity and important in cell senescence. To determine if this activity was essential in the mouse mammary gland *in vivo*, we serially transplanted mammary fragments from wild type (TER^+/+^), heterozygous (TER^+/−^), and homozygous (TER^−/−^) mammary tissues into the cleared mammary fat pads of immune-compromised nude mice. Individual implants from both homozygous and heterozygous TER null outgrowths showed growth senescence beginning at transplant generation two, earlier than implants from TER^+/+^ mammary glands which continued to show growth. This result suggests that either mammary epithelial stem cells maintain their telomere length in order to self renew, or that the absence or reduction of telomerase template results in more frequent death/extinction of stem cells during symmetric divisions. A third possibility is the inability of signaling cells in the niche to replicate resulting in reduction of the maintenance signals necessary for stem cell renewal. Consistent with this, examination of senescent outgrowths revealed the absence of estrogen receptor alpha (ERα^+^) epithelium although progesterone receptor (PR^+^) cells were abundant. Despite their inability to establish mammary growth *in vivo*, TER^+/−^ cells were able to direct neural stem cells to mammary cell fates.

## INTRODUCTION

The action of mammary stem cells and their mitotic progeny is fundamental to normal mammary growth, differentiation, maintenance and regeneration in successive cycles of pregnancy, lactation and involution. An important feature of the mammary gland is the regenerative capacity of its epithelium, which is demonstrated upon successive reproductive cycles. Any portion of the mammary epithelial tree can reproduce the entire functional mammary epithelium upon transplantation into the mammary fat pad [1-5]. Normal mammary transplants to the epithelium-divested mammary fat pad in nulliparous hosts show growth senescence during serial propagation and this was proposed to depend upon the number of mitotic events undertaken by the stem/progenitor epithelial cells [6]. Age and reproductive history do not impact the rate of growth senescence in serial transplantation of fragments or cells in the mammary fat pads [7]. These studies were also performed in parous mice to evaluate the aging process of mammary epithelium [3].

The appearance of growth senescence can be linked to the disruption of the mammary stem cell niche, as not all random fragments chosen reached senescence during the regeneration of the mammary epithelium in any given transplant generation. In cases where fragments continued to grow, we propose that competent stem cell niches must be present. Additionally, we concluded that growth senescence was a direct result of the aging of local mammary stem cells or their niche as they lose their ability to self-renew [8]. The question may be distilled down to stem cell self-renewal. Is this accomplished by symmetric stem cell self-renewal during mammary ductal growth (stem cells divide to give two stem cells) or is the self-renewal of stem cells dependent upon asymmetric cell divisions that are dependent upon signals from a competent non-stem cell niche? We attempt to answer this question by comparing the rate of growth senescence before and after telomere reduction in mammary cells due to generational loss, i.e., by comparing the rate of growth senescence in mammary fragments harvested from generation 1 TER^−/−^ females with those from TER^−/−^ generation 5.

During cellular aging, the repetitive sequences at chromosome ends called telomeres protect intact chromosomes from degradation during successive rounds of cell division. In humans telomeres comprise 10-15 kb and in mice more, 25-50 kb of the repetitive TTAGGG sequences [9, 10]. These repeats progressively shorten with each round of cell division to a point where they no longer can protect the integrity of the chromosomes and instead trigger cellular senescence or apoptosis. To prevent this, cells are able to activate a specific reverse transcriptase enzyme responsible for elongation of telomeric DNA called telomerase (TER). Telomerase is a ribonucleic protein complex that utilizes the protein polymerase actions in concert with a small RNA template known as TERC or TR, that acts as a primer for telomerase DNA extension and “immortalization” of these cells [11, 12]. These ends are then protected by another protein complex termed shelterin to prevent telomere modification by DNA damage surveillance [13]. Telomerase expression is up regulated in germ line cells as well as immortalized cell lines and malignant tumor cells but is repressed in normal human somatic tissues [14, 15].

We hypothesized that regenerative capacity of the mammary gland would be influenced by the presence or absence of telomerase activity since expansion of the stem cell population must occur during branching ductal development in outgrowths. To answer this we utilized a telomerase deficient mouse model in which one or both alleles for the RNA template for the telomerase enzyme have been removed to model a prematurely aged population, to compare growth rate and senescence to that of normal mammary epithelium. Both TER^+/−^ and TER^−/−^ transgenic mice show reduction of telomere length upon successive generations [16]. Telomerase deficient mouse mammary tissues from heterozygous (TER^+/−^) and both first (TER^−/−^g1) and fifth generations (TER^−/−^g5) that have shortened telomeres due to a loss of telomerase activity were transplanted serially in the cleared fat pads of wild type female hosts. As controls, serially transplanted wild type tissues that became growth senescent were analyzed identically to the TER^+/−^ and TER^−/−^ growth senescent implants. To evaluate the capacity of *in vivo*, growth senescent mammary epithelial populations to redirect non-vmammary cells to mammary epithelial cell fates, TER^+/−^ and WAP-TGF-beta1 mammary cell populations were mixed with reporter-marked neural stem cells (NSC). The WAP-TGF-beta1 [17, 18] mammary cells were shown earlier to be growth senescent *in vivo* and their growth senescent ducts were shown to be deficient (lacking) in ER-α^+^ epithelial cells in the present work.

## RESULTS

**Figure 1:**
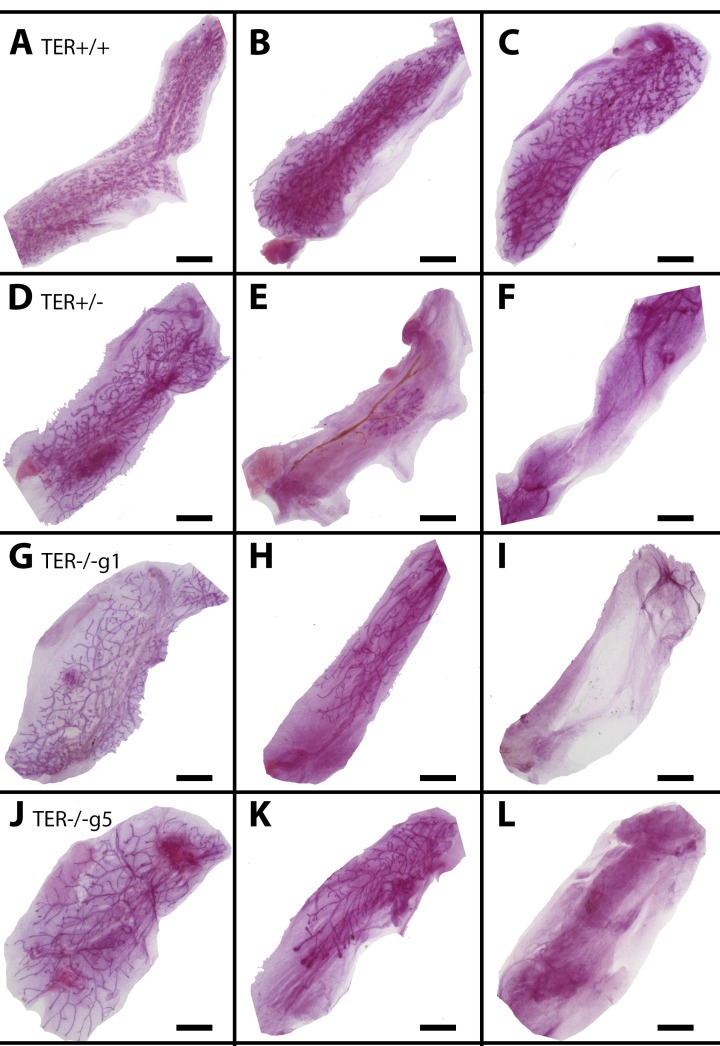
Shortened telomeres lead to enhanced mammary gland senescence upon serial transplantation Whole mounted glands from TER^+/+^ (**A**-**C**), TER^+/−^ (**D**-**F**), and TER^−/−^ (g1 **G**-**I**, g5 **J**-**L**) transplanted tissues from early transplant generations 1 and 2 (A,D,G,J), middle transplant generation 3 (**B**, **E**, **H**, **K**), and late transplant generations 4 and 5 (**C**, **F**, **I**, **L**). Scale bars: 2mm.

To determine the role of reduced telomere length on mammary gland outgrowth, mammary fragments harvested from wild-type (TER^+/+^), heterozygous (TER^+/−^), and telomerase knockout (TER^−/−^) mice were implanted into the cleared inguinal fat pad of three-week old nu/nu female mice and allowed to grow for at least 12 weeks. This initial transplantation yielded a majority of positive takes in all three groups, 20/20, 9/10, and 8/10 respectively. The outgrowths in the wild type and heterozygous groups filled 90-100% of the fat pads and the outgrowths from the knockout group filled similarly albeit two of the transplants grew between 60-80% filling. Fragments were taken from the center of each outgrowth and serially transplanted into new cleared fat pads of three week old mice and then repeated consecutively, thus termed transplant generations two (T gen 2), three (T gen 3), and four (T gen 4). Serially transplanted fragments of wild type epithelium consistently led to positive outgrowths that filled 90-100% of the recipient fat pads (Table 1). In contrast, knocking out one or both copies of the gene encoding the RNA substrate for telomerase gene (TER^+/−^ or TER^−/−^) led to a decrease in positive takes and fat pad filling. Effects could be seen by transplant generation three and maintained through T gen 4 with only 15% of fat pad filling occurring (Figure 1). Thus the absence of telomerase activity and the shortening of mammary epithelial cell telomeres significantly reduced the ability for mammary epithelial growth as compared to wild-type fragments that were able to maintain their chromosomal telomere length.

**Table 1:** Transplantation of telomerase-defective versus wild type mammary fragments in immune-compromised hosts Quantification of serially transplanted TER^+/+^, TER ^+/−^, and TER^−/−^ outgrowths in recipient mice demonstrate a significant decrease in fat pad filling by TER deficient mice as compared to wild type controls. *p <.05

Genotype	T gen 1	T gen 2	T gen 3	T gen 4
**TER^+/+^**	20/20 growths 90-100% filled	19/20 growths 90-100% filled	20/20 growths 90-100% filled	20/20 growths 90-100% filled
**TER^+/−^**	9/10 growths All 90-100%	17/18 growths (16) 70-100% (1) Under 1%	11/20 growths* (1) Ingrowth (3) 50-100% (1) 70% (6) < 50%	8/18 growths* All < 15%
**TER^−/−^ g1**	9/10 growths All 90-100%	14/15 growths All 70-100%	10/10 growths* (3) Ingrowth (4) 50% (3) < 15%	8/10 growths* All < 15%
**TER^−/−^ g5**	8/10 growths (2) 60-80% (6) 100%	13/14 growths (1) Ingrowth (5) 100% (6) 50% (1) < 15%	8/8 growths* (4) Ingrowth (2) 50% (2) < 15%	8/10 growths* (1) Ingrowth (7) < 15%

Telomeres are the repetitive TTAGGG nucleotide sequences found at the ends of each chromatid. Their presence prevents shortening and deterioration during replication of chromosomes necessary for cellular function. The telomeres themselves are maintained by the ribonucleoprotein complex telomerase, which consists of reverse transcriptases known as telomerase reverse transcriptases (TERT). TERT expression is low in most cell types, leading to a progressive shortening of telomere length and subsequent senescence of these cells. To determine whether transplantation of other cells with a senescent phenotype had a similar growth curve as TER null cells, we injected either one hundred thousand TER^+/−^ mammary cells or one hundred thousand WAP-TGF-β1 expressing cells into the cleared fat pad of immune-deficient three-week old female mice. Previously we have demonstrated injection of wild type cells at this concentration consistently leads to full mammary outgrowths, however injection of 100K of either of the senescent cell populations resulted in no mammary outgrowths (Table 2).

**Table 2:** Senescent cell populations reprogram neural stem cells but are not rescued from growth senescence Quantification of serially transplanted TER^+/+^, TER ^+/−^, and TER^−/−^ outgrowths in recipient mice demonstrate a significant decrease in fat pad filling by TER deficient mice as compared to wild type controls. *p <.05

	100k TGF-β1 + 50k Fetal NSCs	100k Wap-Cre/TGF-β1 cells only	50k TER ^+/−^ + 50k Fetal NSCs	100k TER ^+/−^ cells only
1^st^ transplant generation	6/10 positive epithelial growth All positive for x-gal	0/10 No positive takes	6/10 positive epithelial growth 5/6 positive for x-gal	0/10 No positive takes
2^nd^ transplant generation fragments	No positive takes	ND	No positive takes	ND

**Figure 2:**
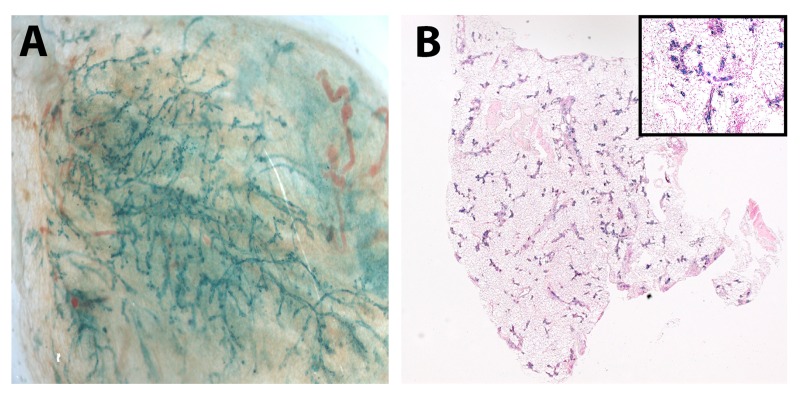
Senescent mammary cells “reprogram” neuronal stem cells in vivo Whole mount picture (**A**) of chimeric outgrowth from TGF-β1^+^ mammary cells (100k) and NSCs (50k) and (**B**) cross section of mammary glands from TER+/− mammary cells (50K) and NSCs (50k). Positive LacZ staining in outgrowths verified NSC incorporation.

While telomerase expression is low in most cell types, it is assumed that stem and progenitor cells maintain telo-merase expression resulting in a protected population capable of giving rise to healthy offspring. To determine if knocking-out TER expression in our mammary stem cells could be rescued we conducted mixing experiments with mouse fetal neural stem cells (NSCs). Briefly, we mixed fifty thousand TER^+/−^ or one hundred thousand WAP-TGF-β1 mouse mammary cells with fifty thousand NSCs and injected them into the cleared fat pads of recipient mice. Interestingly initial outgrowths of these chimeras occurred in six of ten glands by both cell types (Table 2). Presence of NSCs was confirmed by x-gal expression (Figure 2A and 2B).

While mixing WAP-TGF-β1 and TER^+/−^ mammary cells with wild type NSCs resulted in NSC/mammary epithelial hybrid outgrowths, neither the same number of mammary cells from these two populations nor fragment implants from the chimeric outgrowths were able to generate secondary outgrowths when placed into the cleared mammary fat pads of immune-compromised hosts (Table 2). This result demonstrates that the signaling niche required for redirection of non-mammary cells to mammary cell fate was intact in both of these *in vivo* growth-incompetent cell populations and that the NSCs could initially replace the senescent mammary stem cell populations in the majority of recipient glands without compensating for the *in vivo* growth senescence in subsequent transplant gene-rations.

**Figure 3:**
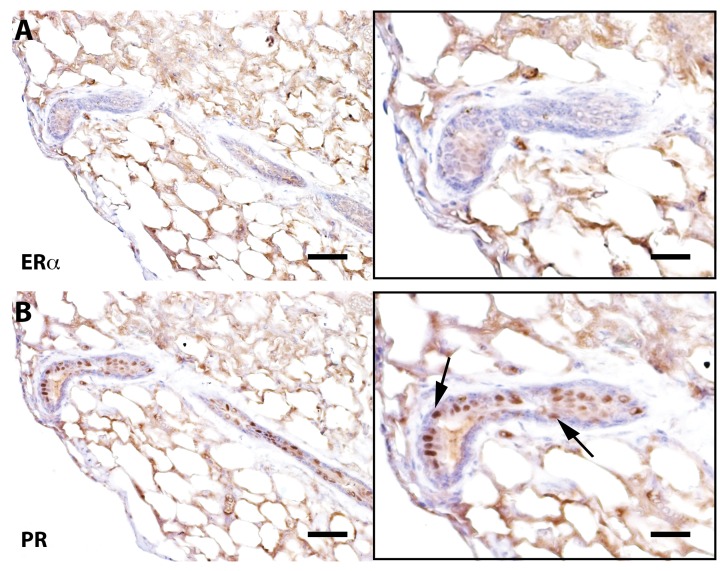
Senescent outgrowths contain PR^+^ cells but lack ER-α^+^ signaling cells Immunohistochemical staining of a cross-section from Wap-Cre/TGF-β1^+^ outgrowths stained for ER-α and progesterone receptor (PR). Arrows denote PR positive cells. Scale bars: 40 μm left panels, 80μm right panels.

Previous work has demonstrated that mammary ductal growth requires estrogen signaling during post-pubertal expansion and that mice lacking estrogen receptor alpha (ERα) specifically, only form rudimentary mammary ducts prior to the onset of puberty [32]. The same authors showed that mixing ERα-null (1:10) mammary cells with wild type mammary cells resulted in the rescue of Erα null cells and both null and wild type mammary cells contributed to positive mammary outgrowths. Further, proliferation of ERα^+^ cells in the mammary gland is controlled by the cytokine TGF-β1 and its overexpression in transgenic mouse models has demonstrated suppression of the proliferation markers Ki-67 and bromodeoxyuridine in ERα^+^ mammary epithelial cells [33]. To determine if early senescence of mammary epithelial cells resulted in a loss of ERα^+^ cells, sections from Wap-Cre/TGF-β1^+^ outgrowths were stained for ERα expression. Corresponding to previous findings, ERα^+^ cells in TGF-β expressing outgrowths were diminished (by ~ 90%) as compared to wild type controls (Figure 3).

## DISCUSSION

Our original premise was to test whether the absence of telomerase activity hastened the loss of the *in vivo* growth capacity of mammary fragment implants compared to wild type. Indeed, mammary fragments from tissue lacking telomerase activity and shortened telomeres showed early growth senescence compared to wild type. These results support the conclusions drawn by Young and Daniel [6] which demonstrated that growth senescence in mammary serial transplants was reached by the number of mitotic events that were required to develop the region of the implant used to regenerate the subsequent transplant demonstration. In other words, implant fragments taken from the center of the outgrowth showed growth senescence less often than implant fragments taken from the periphery of the same outgrowth. In all our serial transplantation experiments the implant fragments were taken from near the center of the outgrowth. Thus TER^+/+^ implants rarely if ever showed growth senescence in four transplantation generations while TER^+/−^ and TER^−/−^ transplant fragments showed early growth senescence despite implant fragments being taken from the outgrowth center of the previous generation.

It is clear from our transplantation results that the regenerative life span of mammary tissue is severely reduced by the absence of telomerase template RNA. The interpretation of this bears careful consideration because the regenerative capacity of mammary epithelium is engendered in the activity of a hierarchy of mammary epithelial stem/progenitor cells and the signals provided by the local cellular environment as well as humoral substances. The question remains whether it is the loss of the mammary epithelial stem cell through symmetric self-renewing divisions or alternatively the loss of its supporting epithelial cell niche that causes the cessation of mammary epithelial growth? It is known that the regenerative capacity of all portions of the mammary epithelial tree is unaffected by age or reproductive status [34]. Therefore, all portions of the mammary epithelial structure must contain mammary stem cells and their niche that retain full regenerative capacity in the intact gland. Why then do portions of the regenerated epithelium lose this ability upon serial transplantation? There are several possibilities. One, the stem cells are exhausted because of repeated self-renewal. Two, the stem cell niche is compromised so that no asymmetric stem cell renewal is possible. Both the stem cell and its niche are compromised, All of these possibilities are the result of symmetric expansion of the epithelial cell whether they belong to the niche or are stem cells. Charles Daniel showed that senescent outgrowths in late generation serial transplants could develop secretory lobules upon pregnancy of the host female [35]. We have asked whether our TER^−/−^ senescent outgrowths repeat these findings. Our attempts demonstrated that no growth could subsequently occur in epithelium-free mammary fat pads implanted with TER^−/−^ or TER^+/−^ fragments from senescent outgrowths and thus no alveolar development was observed. It was reported that no phenotype was observed in the renewing tissues, such as the intestinal, germinal or hematopoietic tissues, in mice null for the telomerase RNA template in early generations, and defects only emerged after four breeding generations [36-38]. This suggests that tissues supported by somatic stem cells that divide asymmetrically and retain their template DNA strands are not affected by the absence of telomerase activity. Interestingly, mammary epithelial fragments from 5^th^ generation TER^−/−^ females did not show more rapid attainment of growth senescence (Table 1) than mammary implants from 1^st^ generation TER^−/−^ females. Thus, telomere shortening in the intact gland does not affect the number of mitotic events necessary to reach growth senescence in serial transplantation.

Telomerase activity has been linked to oncogenesis because of the increased life span of carcinogenic tissues and cells [39]. Further the loss (deletion) of the TER RNA substrate has recently been associated with a reduction of tumorigenesis in endocrine tissue in vivo in a transgenic mouse model [40-43]. The effects of the loss of telomerase activity in regenerating mammary tissue has not been reported earlier, This report indicates that the maintenance of growth potential in serial transplanted mammary tissue is dependent upon telomerase activity. However, telomerase activity is apparently dispensable during morphogenesis of mammary tissue because there was no reduction of regenerative capacity when random fragments were transplanted from late generation TER^−/−^ females. It was shown earlier that haplo-insufficiency in TER^+/−^ mice was sufficient for telomere shortening [44], The demonstration of early senescence in serially transplanted TER^+/−^ mammary fragment implants indicates telomere shortening is the likely mechanism for early growth senescence in TER deleted mammary outgrowths.

It is somewhat more difficult to explain why or how mixing non-mammary cells with mammary cells incapable of *in vivo* growth by themselves results in the production of chimeric outgrowths composed of cells from both populations. It is tempting to conclude that the non-mammary cells upon redirection to mammary epithelial cell fate supply the missing component(s) required for in vivo growth, yet fail to maintain this property in subsequent transplant generations. Examination of the positive outgrowths obtained when mixing the neural stem cells with TER^+/−^ and WAP-TGFbeta1 mammary cells indicate that no ER-alpha positive cells are present. This could be an explanation for the failure of secondary outgrowths from transplants of these tissues. Nevertheless, the non-mammary cells were redirected to mammary epithelial cell fates and in combination with the growth-deficient mammary cells produced robust primary outgrowths in post-partum females.

We conclude that the increase in reaching growth senescence in the mammary epithelial transplants is the result of telomere shortening, this must occur during the development of the outgrowth from the implanted fragment. No decrease in the rate of positive takes or the amount of implanted fat pad filled in the early passages is observed even in implants from generation #5 TER^−/−^ females. This suggests that cell division as demonstrated by Daniel and Young in 1971 [6] is paramount in the appearance of growth senescence. Is this due to symmetric expansion of the mammary epithelial stem cell or the telomere shortening suffered by the epithelial cells forming the niche? Our immunochemical evidence implicates the absence of ERα-positive epithelial cells and cessation of ductal growth. This is in accordance with the absence of post-pubertal mammary duct expansion in ERα-null female mice.

## CONCLUSIONS

It appears that estrogen responsive cells (ERα^+^) are required for continued *in vivo* proliferation in post-pubertal female hosts. Growth senescent outgrowths (unable to produce ductal elongation) contain PR^+^ cell and can respond to pregnancy by producing lobulo-alveolar structures that express milk proteins (Fig. Supplemental 1). Cells, which are unable to produce outgrowths, *in vivo*, are growth competent when mixed with non-mammary neural stem cells. Examination of these outgrowths indicated that they were devoid of ER-α^+^ epithelial cells. Accordingly, these outgrowths fail to sustain the ability to produce secondary epithelial outgrowths when transplanted.

All senescent outgrowths, irrespective of their telomerase activity appear to be devoid of ER-alpha-positive mammary epithelial cells. This was also true of the dispersed mammary epithelial populations (Table 2) that were unable to grow successfully *in vivo*. Despite this, the senescent mammary epithelial populations in Table 2 were able to redirect non-mammary cells to attain mammary epithelial cell fates when injected together and turn contributed to mammary growth *in vivo*.

## MATERIALS AND METHODS

### Ethics statement

Investigation has been conducted in accordance with the ethical standards and according to the Declaration of Helsinki and according to national and international guidelines and has been approved by the authors' institutional review board.

### Mice

Female Athymic NCr Nu/Nu mice were used for transplantation studies. TER wild type, TER^+/−^, and TER^−/−^ mice were generously gifted from Dr. Richard J. Hodes (CCR, NCI) and have been previously described [19]. WAP/TGF-β 1^+^ mice were engineered as previously described [20]. All mice were housed in Association for Assessment and Accreditation of Laboratory Animal Care-accredited facilities in accordance with the National Institutes of Health Guide for the Care and Use of Laboratory Animals. The National Cancer Institute (NCI) Animal Care and Use Committee approved of all experimental procedures.

### Mammary fat-pad clearing

The surgical procedures for clearing the mammary epithelium from the #4 inguinal fat pads of 3 week-old female mice and the method of implanting either tissue fragments or cell suspensions have been described in detail in earlier publications [21-26]. Generally, the surgical procedures required to remove the host epithelium from the fat pads were performed immediately prior to insertion of the transplant or inoculation of cultured cells.

### Tissue transplantation and cellular inoculation

Random fragments (∼1.0 mm3) of mammary epithelium were taken from virgin female glands. The fragments were implanted as described above and the hosts were bred after 3 weeks. The implanted glands as well as host glands were taken at 8 weeks post-op. Fragments were obtained from the outgrowths to be used in subsequent transplant generations. Eight to ten fragments were selected at random from the glands. This procedure was repeated for 5 transplant generations or until growth senescence was achieved. Alternatively cell suspensions were injected in a ratio of 100,000 cells/10μl volume of non-supplemented Dulbecco's Modified Eagle's Medium (DMEM) or sterile phosphate-buffered saline, pH 7.2 with a Hamilton syringe equipped with a 30 gauge needle.

### Mammary epithelial cell dissociation

Mammary glands were harvested and dissociated with 0.1% collagenase (Sigma) overnight at 37°C in complete tissue culture medium with 10% fetal calf serum. The following day samples were triturated with a 10 mL sterile pipette and passed through a 19-gauge needle. Resulting organoids were cultured in plastic flasks in DMEM supplemented with 10% fetal bovine serum, insulin (1.0 μg/ml), and epidermal growth factor (10 ng/ml). Fibroblasts were removed by differential trypsinization after 72 hours and mammary epithelial cells collected 24 hours later.

### Neural Stem Cell (NSC) isolation

NSC isolation was performed as previously described [27]. Isolation of NSCs was performed using an established protocol, based on selective expansion, for the isolation and expansion of NSCs from the fetal and adult mice brains. After dissection, triturated tissue is plated in a culture medium that supports the expansion of stem cells but not other cell types. Cells are cultured in a medium composed of apotranferrin (for iron transport), insulin (as a pro-survival signal), and basic fibroblast growth factor (mitogenic for NSCs) [28].

Fetal cultures are normally passaged approximately every 5 days. After the first passage (which removes most of the remaining contaminants), the cultures are composed of 95% NSCs. Clonal and real-time lineage analyses confirm their self-renewal properties and multipotential. Adult cultures are composed of both multipotent [28, 29] stem cells and a glial-restricted progenitor, but their morphologies are distinct, and because they are cultured under clonal conditions, they are easily distinguished.

Fetal cultures are derived from E13.5 mouse embryo cortex, whereas adult cultures are derived from the sub ventricular zone of 2- to 3-month-old mice. After dissection, the culture conditions are identical for these two stem cell sources. Cells were then maintained in culture as previously described [27].

### Preparation of mammary gland whole mounts and X-gal staining

Briefly, the number #4 inguinal fat pads were excised from the transplant-bearing mice and spread onto glass slides. The glands were spread to expose as much area to the glass and to flatten the sample to improve viewing. The glands were then fixed in Carnoy's fixative (1:3:6 ratio of acetic acid, chloroform and ethanol) for 4 h at room temperature. They were then stained with carmine alum, dehydrated through a series of alcohols, cleared in xylene, and sealed with Permount and a glass coverslip. Whole mounts of inguinal glands containing LacZ^+^ cells were fixed and stained as previously described [22, 30]. Briefly, whole inguinal fat pads were mounted on glass slides and fixed in 4% paraformaldehyde for 2 hours, per-meabilized in 0.02% NP-40, 0.01% sodium deoxycholate, and 0.002 M MgCl_2_ in phosphate-buffered saline (1X PBS) overnight at 4°C and processed for X-gal as previously described [31]. For X-gal controls intact host gland were treated identically. Stained glands were repeatedly rinsed in 1X PBS, then post fixed with Carnoy's fixative. Glands were then dehydrated in a graded series of alcohol and cleared in xylenes before analysis.

### Immunohistochemistry

For histological examination, X-gal + glands were embedded in paraffin and cut in 5 μm sections and mounted on positively charged slides. Sections were subsequently cleared in xylenes and rehydrated through ethanol gradients. Antigen retrieval was performed by heating slides in a boiling water bath for 20 min. in either 10mM citrate buffer pH 6.0 (Dako, Capenteria, CA) or Tris-EDTA pH 9.0 (Dako). Endogenous peroxidase activity was blocked by using 3% hydrogen peroxide for 15 minutes at room temperature. Slides were blocked with normal horse serum (Vector Laboratories, Burlingame, CA) for 1 hour at room temperature and then incubated overnight with primary antibodies at 4°C. Primary antibodies used were rabbit anti-PR (1:150; Dako), rabbit anti-ERα sc#-542 (1:75; Santa Cruz Biotechnology, Dallas, TX). Secondary antibody staining was performed using the R.T.U. Vectastain (goat anti-rabbit/mouse) kit (Vector Laboratories). Staining was visualized using the DAB peroxidase substrate kit (Vector Laboratories) per manufacturer's recommendations. Slides were counterstained with Mayer's hematoxylin (Sigma-Aldrich) and negative tissue controls were included in all immunohistochemical analyses.

## SUPPLEMENTAL DATA FIGURE


