# Supplementary material for: Telomerase and estrogen-sensing activities are essential for continued mammary growth in vivo but dispensable for “reprogramming” neural stem cells
Source: Aging (Albany NY). 2016 Jun 24;8(7):1353–62. doi: 10.18632/aging.100985 (PMC4993335; doi:10.18632/aging.100985)
Supplement: Supplementary file 1 [file aging-08-1353-s001.pdf]

SUPPLEMENTAL DATA

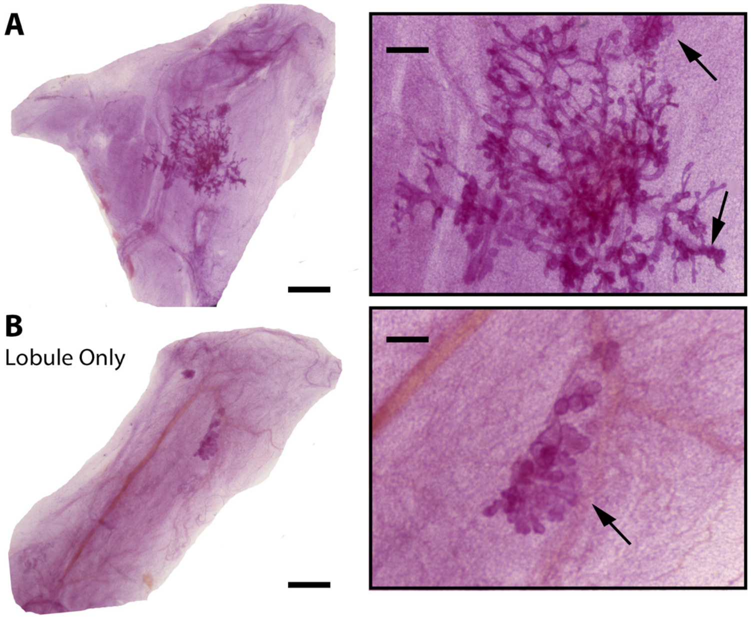

Supplemental Figure 1. Mammary outgrowths from wild type senescing populations are capable of forming lobules during pregnancy. Stained whole mounts from wild type outgrowths at parturition demonstrate ductal and lobule (**A**) and lobule-only (**B**) growth during pregnancy. Arrows denote lobule structures. Scale bars: 2mm left panels, 500µm right panels
